# Anatomy in the animal kingdom

**DOI:** 10.1038/s42003-021-02613-0

**Published:** 2021-09-15

## Abstract

Examining the anatomy of an organism opens up a whole world of exploration into the function of its body, its evolution, and how it interacts with the biotic and abiotic elements in its environment. On the cusp of new advances in technology that have furthered this exploration, the editors at *Communications Biology* have gathered a Collection of our exciting research in organismal anatomy to highlight the possibilities of this field yet to come.

Data exploration is the initial step of any new scientific analysis. Getting a “feel” for your data can inform and direct more formalised statistical approaches, or encourage future experiments. When considering organismal biology, the anatomy of the animal, plant or microbe is the data, and exploration happens through observations.

For hundreds of years, biologists have been using anatomical insight to learn more about the world surrounding us. Some of the most influential scientific discoveries of the past are based on observing organism anatomy--the basis for the evolution by natural selection popularised by Charles Darwin^1^ (with due credit to Alfred Russel Wallace), Owen’s naming of Dinosauria^2^, or Mendel’s demonstration of genetic inheritance. Without detailed, insightful observations of the world around them, these discoveries could not be made.

For hundreds of years, biologists have been using anatomical insight to learn more about the world surrounding us. Some of the most influential scientific discoveries of the past are based on observing organism anatomy.

Whilst the most immediate observations might occur on a macroscopic level, such as examining a fossil or living creature in its natural habitat, anatomical observations are not limited to this wide lens. Using modern advances in technology, we can look beyond the surface level structures of an organism, delve deeper into its anatomy, and make inferences about the evolution of its function. Computed tomography, scanning electron microscopy or synchrotron scanning are becoming commonplace methods in anatomical research in observing organisms from both modern and deep time. Combinations of these methods are enabling researchers to ask questions that were previously unanswerable, such as the fine details of venom delivery in snakes^3^, or the importance of fully understanding anatomical constraint before inferring function in fossil vertebrates^4^.

*Communications Biology* appreciates the value of anatomical research in the modern age, and has created a collection of some of our published manuscripts highlighting anatomical structures and the inferences they yield. Our published papers span palaeontological studies enabling phylogenetic and functional inference, studies of extant systems with significant genetic or ecological context, and structural analyses of microscopic details focussing on the biological function of organisms, plus many more assembled together by our editors in our Anatomy Collection. We welcome further submissions featuring anatomical research across all species, especially for those highlighting functional or ecological analyses.

**Figure:**
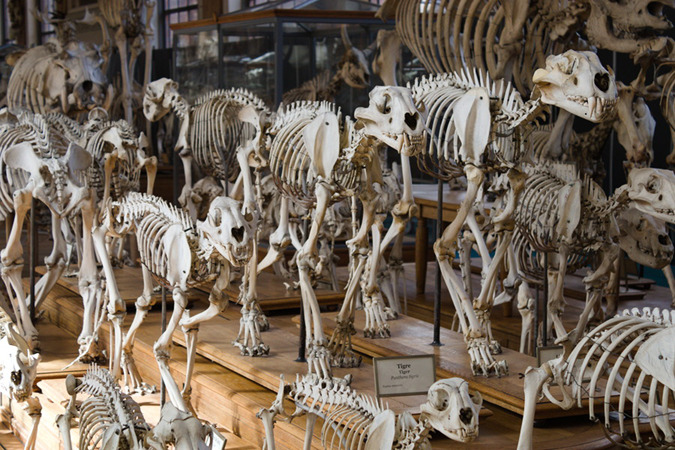
Luke R. Grinham
